# Norovirus infections and knowledge, attitudes and practices in food safety among food handlers in an informal urban settlement, Kenya 2017

**DOI:** 10.1186/s12889-020-8401-x

**Published:** 2020-04-10

**Authors:** Eliud Wainaina, Christina A. Otieno, Joseph Kamau, Atunga Nyachieo, Sara A. Lowther

**Affiliations:** 1 Moi University, Eldoret, Kenya; 2 Field Epidemiology and Laboratory Training Program, Ministry of Health Program, Kenyatta National Hospital Grounds, P.O Box 22313–00100, Nairobi, Kenya; 3 Institute of Primate Research, Karen, Kenya; 4 University of Nairobi, Nairobi, Kenya; 5 Centers for Disease Control and Prevention, Atlanta, GA USA

**Keywords:** *Norovirus*, Food handlers, Informal settlement, Kenya

## Abstract

**Introduction:**

A leading cause of acute gastroenteritis, norovirus can be transmitted by infected food handlers but norovirus outbreaks are not routinely investigated in Kenya. We estimated norovirus prevalence and associated factors among food handlers in an informal urban settlement in Nairobi, Kenya.

**Methods:**

We conducted a cross-sectional survey among food handlers using pretested questionnaires and collected stool specimens from food handlers which were analyzed for norovirus by conventional PCR. We observed practices that allow norovirus transmission and surveyed respondents on knowledge, attitudes, and practices in food safety. We calculated odd ratios (OR) with 95% confidence intervals (CI) to identify factors associated with norovirus infection. Variables with *p* < 0.05 were included in multivariate logistic regression analysis to calculate adjusted OR and 95% CI.

**Results:**

Of samples from 283 respondents, 43 (15.2%) tested positive for norovirus. Factors associated with norovirus detection were: reporting diarrhea and vomiting within the previous month (AOR = 5.7, 95% CI = 1.2–27.4), not knowing aerosols from infected persons can contaminate food (AOR = 6.5, 95% CI = 1.1–37.5), not knowing that a dirty chopping board can contaminate food (AOR = 26.1, 95% CI = 1.6–416.7), observing respondents touching food bare-handed (AOR = 3.7, 95% CI = 1.5–11.1), and working in premises without hand washing services (AOR = 20, 95% CI = 3.4–100.0).

**Conclusion:**

The norovirus infection was prevalent amongst food handlers and factors associated with infection were based on knowledge and practices of food hygiene. We recommend increased hygiene training and introduce more routine inclusion of norovirus testing in outbreaks in Kenya.

## Introduction

Worldwide, foodborne illness causes considerable morbidity and mortality [1] as well as billions of dollars in healthcare-related costs each year [2]. Norovirus, the leading cause of foodborne illness worldwide, is frequently transmitted by food handlers [3], and is a major cause of acute gastroenteritis among persons of all ages globally [1]. Norovirus is a small, non-enveloped single-stranded RNA viruses that typically cause illness 10–51 h after ingestion of a relatively low number of viral particles, with symptoms that include acute-onset nausea, vomiting, watery diarrhoea, stomach cramps, headache and fever [4]. While norovirus is documented as a leading cause of foodborne diseases globally [1], the epidemiology of norovirus is poorly characterized in Kenya.

Typically transmitted by fecal-oral spread, norovirus can enter the food chain at multiple points, but most commonly when food products, water, or fomites are contaminated by infected food handlers [3]. When food is the vehicle of transmission, contamination occurs most often through improper handling of food directly before consumption [3]. However, norovirus contamination has occurred further upstream in the food chain, such as one instance of contamination of prepackaged delicatessen meat [5].

Understanding food safety procedures and potential causes of foodborne illness are important for all food handlers. “Ready-to-eat” food and food eaten raw can be contaminated by preparers’ hands, by raw food ingredients [6], or by contaminated environmental surfaces [7]. For disease prevention, Kenyan law states that food products should be prepared hygienically to avoid contamination, and requires all food handlers undergo regular medical screening to prevent communicable disease transmission through food [8], which results in a medical certificate allowing them to sell food products. Lacking widespread availability of norovirus testing, norovirus screening is not part of current medical examination screening for Kenyan food handlers, [9] which focuses on bacterial and parasitic causes of foodborne illness.

In an informal settlement in Nairobi, Kenya, previous studies have identified norovirus as a cause of illness among ill persons [10] in the community, as well as in environmental water [11]. Infections with norovirus among food handlers in this informal settlement have not been investigated. Therefore, we sought to estimate norovirus prevalence among food handlers, and assess the knowledge, attitudes, and practices in food hygiene among food handlers in this informal settlement.

## Methods

The setting for this study was in an informal urban settlement in Nairobi, Kenya, which has an estimated 300,000 inhabitants and population density of 49,228 persons per square kilometer according to Kenya National Bureau of Statistics (2009). The area’s health system is organized through 13 community health units of varying population size. A study on sanitation in this area found that, among households surveyed, 99% did not have access to improved sanitation facilities (flush or pour toilet or latrine connected to sewer, septic tank, or pit), 82% accessed shared sanitation facilities, 14% accessed unimproved sanitation facilities, and 3% of households practiced open defecation (i.e., no sanitation facilities) [12].

We conducted a cross-sectional survey among food handlers in this informal urban settlement between 12 April and 27 June 2017. For this study, we included all premises where unpacked, locally prepared, ready-to-eat foods were sold directly to the public within the study area. This included open places either by the roadside or in a market, structured kiosks and buildings, or in mobile pushcarts. Any food handler aged 18 years or older capable of responding to interview questions that was involved in the sale of ready-to-eat unpacked food, prepared at home or on any establishment within the study area, was eligible for the study. Although the main focus of this study was on food, we also asked about provision of clean water for drinking, hand washing, or other food preparation steps.

Using the Cochran formula [13] and assuming a prevalence of 10% [14] among a population of a total of 2720 food handlers and precision of 0.035, we estimated the minimum sample size to be 303 food handlers after adjusting for 10% non-response. To enroll a representative sample of food handlers, we first enumerated the total number of food handlers in the area by consulting trained community health extension workers (CHEWs) and calculated a sampling interval of every tenth food handler. We then divided up the area into its constituent community health units (CU) and selected 1 in 10 food handlers in that health unit by assigning each an ID on a paper, placing in a basket, and picking the appropriate number of food handlers for that health unit with no replacement.

Following an informed verbal consent process, we administered pre-tested questionnaires (Supplementary information) through in-person interviews, collecting demographic information such as age, sex, and highest educational level. We also recorded clinical information on diarrhoea or vomiting during the previous month. We did not inquire about active gastrointestinal illness out of concern that this would (a) decrease response rates and (b) not be accurately reported out of food handlers’ fear of persecution for working while ill. We recorded information on knowledge, attitudes, and practices from the food handlers on food and water safety and hygiene. CHEWs assisted us to carry out an observational checklist that collected information about hygienic practices before, during, and after the interview.

Trained community health volunteers (CHV) instructed each respondent on the specimen collection procedure at their home while the trained CHEWs helped with questionnaire administration. We labelled the stool specimen container with a unique identification number after recording in a register, and included each participant’s geographical work zone (wards), CU name, and date of collection. The enrolled food handler was trained on specimen collection whereby about two to 3 grams of stool was collected in a wide sterile-mouthed container, which was immediately put in a portable liquid nitrogen gas container at -196 °C for transportation purposes. We transported stool specimens within 6 hours of collection to the Kenya Institute of Primate Research laboratories for norovirus detection and storage purposes.

We extracted RNA in the laboratory using phenol chloroform as previously described, [15] followed by conversion into copy DNA(cDNA) using the Thermo Scientific™ Rivert Aid kit. We used genogroup I and II norovirus primers (Liferiver Biotechnology Company, Shanghai, China) as described in the past [16], and the Invitrogen™ Platinum kit for polymerase chain reaction (PCR). The PCR amplification conditions were denaturisation (95 °C), annealing (55 °C) and extension (72 °C) for 35 cycles. After testing, the specimens were preserved at -80 °C.

We calculated descriptive statistics for characteristics of the respondents and answers from their knowledge, attitudes, and practices. To consolidate responses relating to food handlers’ attitudes, we grouped responses related to financial gain or loss as “economic purposes”, and responses given by food handlers related to maintaining or disrupting cleanliness as “hygienic purposes”. All responses related to the physical or mental well-being of a person were grouped as “health reasons”.

We used the Pearson chi-square test to examine the association between dependent and independent variables, and identify potential confounders or effect modifiers. Crude odds ratios (OR) and 95% confidence intervals (CI) were calculated to examine the relationship between different characteristics and testing positive for norovirus infection. Significant risk factors (*p* < 0.05) in crude analysis were incorporated in multivariable logistic regression models using step-wise backward approach. To adjust for factors simultaneously, we calculated adjusted odds ratio (AOR) and 95% CI.

Our study was approved by the Moi University Institutional Research and Ethics Committee, Eldoret, Kenya (Formal approval number FAN: IREC 1853), and by the Kenya National Commission for Science and Technology (NACOSTI) permit number: NACOSTI/P/17/33581/15836. The survey was determined to be non-research by the U.S. Centers for Disease Control and Prevention, Centers for Global Health.

## Results

We approached 303 food handlers for participation; 20 (7%) persons declined, and therefore 283 food handlers completed the interview. The 283 study respondents had a mean age of 33.4 years (range, 18–64 years) (Table 1). Most enrolled food handlers (47%) sold food from mobile structures. The food handlers most frequently had secondary education (47%), and women comprised the majority of respondents (70%).

**Table 1:** Food handler’s socio-demographic characteristics in relation to norovirus infection status in Kibera, Kenya 2017

Variables (***n*** = 283)	norovirus Positive		norovirus Negative		Odds Ratio	95%Confidence Interval (CI)	
	***N*** = 43	%	***N*** = 240	%	(OR)	Lower	Upper
**Age (years)**							
18–39	31	14	190	86	Ref	–	–
40–64	12	19.4	50	80.6	1.47	0.70	3.6
**Gender**							
Female	31	15.7	166	84.3	Ref	–	–
Male	12	14	74	86	0.87	0.42	1.78
**Education level**							
Primary	24	19.2	101	80.8	Ref	–	–
Secondary	17	12.6	118	87.4	0.61	0.11	9.84
Tertiary	1	5.6	17	94.4	0.25	0.31	1.19
None	1	20	4	80	1.05	0.03	1.95
Income per month*							
< 5000	17	19.1	72	80.9	Ref	–	–
5001–1000	14	15.7	75	84.3	0.79	0.36	1.72
1001–15000	4	7.3	51	92.7	0.33	0.11	1.05
> 15001	8	16	42	84	0.81	0.32	2.03
**Premise type**							
None	3	8.3	33	91.7	Ref	–	–
Fixed	17	14.9	97	85.1	1.93	0.53	7.00
Mobile	23	17.3	110	82.7	2.3	0.65	8.14
**Wards**							
*Makina*	12	20.3	47	79.7	Ref	–	–
*Sarangombe*	10	14.5	59	85.5	0.66	0.26	1.67
*Lindi*	9	19.2	38	80.9	0.93	0.35	2.43
*Laini Saba*	4	9.3	39	90.7	0.40	0.12	1.35
*High Rise*	8	12.3	57	87.7	0.55	0.21	1.46

* Kenyan shillings *Ref Reference

Of the 283 respondents, 43(15.2%) stool specimens were positive for norovirus PCR. Among the socio-demographic characteristics analyzed (age, gender, education, income level, type of premise, and wards of residence), none were associated with norovirus positivity (Table 1). Respondents who reported both diarrhoea and vomiting in the past month were more likely to have norovirus infection compared to those who reported neither diarrhea nor vomiting (OR = 4.6, 95% CI = 1.9–11.0). Reporting that a household member had both diarrhoea and vomiting in the past month was also associated with norovirus positivity in the food handler (OR = 6.7, 95% CI = 2.6–17.5 (Table 2).

**Table 2:** Health status on diarrhea and vomiting among food handlers and their households in an informal urban settlement, Kenya, 2017

Variables	norovirus Positive		norovirus Negative		OR	95%CI	
	n	%	n	%		Lower	Upper
**Respondents’ knowledge on that local environment can contaminate ready-to-eat food**							
Yes can contaminate	37	14	23	86	Ref	–	–
No can’t contaminate	2	20	8	80	1.54	0.31	7.54
Don’t know answer	4	50	4	50	**6.16**	**1.48**	**25.72**
**Respondents’ knowledge that spitting on drinking water can contaminate ready-to-eat food**							
Yes can contaminate	36	14.3	216	85.7	Ref	–	–
No can’t contaminate	6	40	9	60	**3.97**	**1.34**	**11.92**
Don’t know answer	1	6.3	15	93.7	0.40	0.05	3.12
**Soap and water availability at the point of sale as observed**							
Yes both available	12	7.5	149	92.5	Ref	**–**	**–**
No not available	31	25.4	91	74.6	**4.17**	**2.08**	**8.33**
**Household member with diarrhea**							
Yes	11	34.4	21	65.6	**3.57**	**1.59**	**8.33**
No	32	12.7	219	87.3	Ref	–	–
**Household member with vomiting**							
Yes	12	37.5	20	62.5	**5.26**	**2.13**	**12.5**
No	31	12.4	220	87.6	Ref	–	–
**Household member with**							
*Neither Diarrhoea/ Vomit*	32	12.9	216	87.1	Ref	–	–
*Only Diarrhoea*	1	8.3	11	91.7	0.61	0.08	4.91
*Only Vomit*	0	0	3	100	UD*	UD	UD
*Both Diarrhoea and Vomit*	10	50	10	50	**6.75**	**2.61**	**17.49**

*UD Undefined, *Ref Reference

Most food handlers surveyed had basic knowledge on the rationale for food hygiene. More than 90% of the enrolled food handlers had the basic knowledge that hands should be washed before handling ready-to-eat food to prevent illness. When asked to agree or disagree with ways that food can be contaminated after cooking, having a dirty chopping board was mentioned by 87% of food handlers, while vomit aerosols from infected persons was named by 83% of respondents. Nearly all (97%) thought that a dirty environmental location of sales could contaminate ready-to-eat food, and 93% believed that spitting in drinking water could cause contamination.

Of the 283 respondents, 19 (7%) stated that they had a medical examination certificate, required to sell food, but only eight medical certificates were physically observed. Food handlers reported the two most important reasons for having an official medical examination certificate before selling food were overall self-awareness of health status (85%) and the ability to prevent disease transmission (82%). Regarding attitudes towards food handling, 84% agreed that they should obtain a medical examination certificate before engaging in ready to eat food business while 2% disagreed; 14% did not know they needed a medical examination certificate before engaging in business. Reasons given by food handlers who believed they should obtain medical examination certificates included: health purposes (63%), law compliance (19%), to ensure good relations with health officials (15%), and economic reasons (4%). Erroneous reasons given by those who said that a medical examination certificate was not important (14%) were because they worked on part-time basis (76%), and the need for medical examination certificates is based on the type of food one is selling (24%).

Most food handlers held attitudes and beliefs that corresponded to ensuring food safety for their customers. Nearly all (97%) food handlers believed that all food handlers were responsible for washing their hands before food preparation. Reasons given for hand washing included to prevent diseases (59%), hygiene purposes (39%), and economic purposes (2%). Explanations given by those who believed that food handlers were not required to wash their hands included that it depended on type of food sold (50%), how busy the food handler was (33%), and availability of water for hand washing (18%). Respondents believed that food handlers should worry about diarrhea among themselves for health reasons (79%) and economic reasons (21%). Among seven (4%) respondents who did not believe that food handlers should worry about diarrheal illness, reasons given included that they take monthly medication to prevent illness (2 respondents), they believe food is always safe (2 respondents), they believed that they cannot infect customers (3 respondents), and they do not receive complaints from customers and so do not worry about it (3 respondents). Only 2.5% of respondents believed that it was not a food handler’s duty to ensure that water safe for drinking was served to their customers. However, very few had specifically heard about norovirus: of the 32 (11%) food handlers who reported that they had heard about norovirus, 19 (6.7%) had learned of norovirus from the media, 8 (2.8%) had heard of it from health personnel and 5 (1.8%) did not recall where they had heard of it.

Improvement of food safety practices was the reason given by 95% of participants for why food handlers should undergo food hygiene training. Reasons for training to improve food safety were health and disease prevention (63%), general skills and acquired experience to maintain standards (27%), and economic reasons related to maintaining financial gain (10%). Among the 6% of respondents who did not believe that training was required for food safety practices, they felt that training was a waste of time (33%), experience was better (27%), knowledge was inborn/innate (20%), and because food handlers worked part-time (20%). Past formal training on food safety was reported by 12% of respondents.

Practices reported towards diarrhea prevention at their workplace by participants included hand washing regularly (98%), avoiding food handling with bare hands (96%), not allowing customers to select food with bare hands (96%), not handling food while sick with diarrhea (95%), washing the cutting board after using (91%), and taking medication to prevent and treat diarrheal illness (90%).

However, we observed some differences in practices compared to these reported practices. During observation, out of 283 respondents, only a quarter (26%) of food handlers were observed hand washing before handling ready-to-eat food, and 40% were observed touching ready-to-eat food with bare hands. We found that 43% worked in premises without soap and water for hand washing. The study team observed 39% of respondents blowing inside polythene paper to open the bags when serving food to their customers. We rarely observed (3%) raw food in contact with ready-to-eat food.

Among the respondents’ responses regarding to knowledge, attitudes, and practices, and from the interviewer’s observational checklist, several factors were related to testing positive for norovirus in the bivariate analysis (Table 2). Factors which remained independently associated with finding norovirus in stool in the multivariate model were: reporting diarrhea and vomiting within the previous month (AOR = 5.7, 95% CI = 1.2–27.4), not knowing aerosols from infected persons can contaminate food (AOR = 6.5, 95% CI = 1.1–37.5), not knowing that a dirty chopping board can contaminate food (AOR = 26.1, 95% CI = 1.6–416.7), respondents observed touching food bare-handed (AOR = 3.7, 95% CI = 1.5–11.1), and working in premises without hand washing services (AOR = 20, 95% CI = 3.4–100.0) (Table 3).

**Table 3:** Crude and adjusted factors associated with norovirus infection among food handlers in Kibera informal settlement 2017

Variables	positive	negative	COR	AOR	95%CI	
**Respondent symptom**						
Neither Diarrhea/Vomit	27	210	Ref	Ref	–	–
Only Diarrhea	4	10	3.11	11.95	1.70	84.09
Only Vomit	2	3	5.19	13.21	0.63	275.12
Both Diarrhea/Vomit	10	17	4.58	5.69	1.18	27.36
**Participants’ knowledge that dirty chopping board can contaminate food**						
Yes	28	218	Ref	Ref	–	–
No	6	15	3.11	2.39	0.30	19.23
Don’t Know	9	7	10.01	41.72	2.51	694.07
**Participant knowledge that aerosols can contaminate cooked food**						
Yes	25	210	Ref	Ref	–	–
No	10	22	3.82	9.39	1.74	50.57
Don’t Know	8	8	8.40	1.31	0.08	21.95
**Observed Hand washing services availability at the point of sale**						
Available	17	185	Ref	Ref	–	–
Not available	26	55	5.00	17.51	3.12	98.27
**Food handler observed touching ready to eat food bare handed**						
No	18	152	Ref	Ref	–	–
Yes	25	88	2.38	3.03	1.03	8.88

*Ref Reference

## Discussion

In this low-income, urban, highly populated informal settlement, norovirus infection was common among food handlers. Gaps were found in the food handlers’ knowledge and practices regarding food safety and food hygiene, and some of these factors related to hygiene were also associated with norovirus infection. Specifically, these factors include food handlers who reported symptoms consistent with norovirus in the past month, lack knowledge or had misconceptions about food contamination, work in premises without hand washing services, and were observed touching ready-to-eat food. The lack of hand washing services likely leads to less frequent hand washing among food preparers, which could lead to a greater likelihood of norovirus infections. Although knowledge on food safety does not always translate into practice, we found that the lack of knowledge on disease transmission might indicate generally unhygienic practices that could have exposed food handlers to norovirus infection. Ready-to-eat food can easily be contaminated by preparers’ hands [7]; therefore this practice poses a risk of norovirus to customers of food handlers with poor practice.

Seventy-four percent (32/43) of participants that tested positive for norovirus reported no diarrhea or vomiting in the previous month, indicating either asymptomatic infections in the study area, or that food handlers were reticent to disclose recent gastrointestinal illness to the survey team. Norovirus-positive asymptomatic food handlers have been documented elsewhere [3] with norovirus-infected food handlers contaminating ready-to-eat food being the most common source of foodborne norovirus outbreaks [3]. Household norovirus transmission might be reflected based on reports of symptomatic family members, which has been documented elsewhere [17]. Our study did not test for other causes of diarrhea or vomiting. Furthermore, norovirus detection may have been underestimated given the assay used, instead of a more sensitive real-time RT-PCR.

Both the Food and Agriculture Organization and the World Health Organization recommend that food handlers undergo training before they are licensed to do business, as inadequate food handling practices pose a threat to food safety [18]. This is also recommended in Kenya [8]. However, only 12% of food handlers surveyed reported having undergone food hygiene training. Globally, outbreaks have occurred when untrained food handlers were working while symptomatic with norovirus [19], however norovirus outbreaks have not been investigated in Kenya. We found lack of compliance with the existing food safety laws, with very few food handlers having a medical certificate indicating previous training. Increasing compliance with hand washing can reduce norovirus infection [20], and hand washing with soap is most effective in removing contamination with viral pathogens [21].

One strength of our study was that it involved not only reported knowledge, attitudes, and practices by food handlers, but also observations to confirm reporting. However, our study was limited because it only included observations at the point of sale, while some food processing that occurred at home may have identified other unsanitary food handling practices. Food handlers are also likely to over-report and over-perform good behaviors during the study compared to their usual practices when not observed. The norovirus prevalence might have been underestimated if food handlers who were ill at the time of the study enrollment avoided participation because they feared that study participation might have impacted their sales. However, only 20 food handlers declined participation, and therefore we do not expect a major impact to our findings. Our study may also not accurately represent all food handlers in the study area; however, we did systematically enroll food handlers through community members who were familiar with the area with limited non-response. Finally, limited funding availability did not permit us to sequence norovirus for confirmation.

We recommend better characterization of the contribution of norovirus in outbreaks of acute gastroenteritis by including norovirus testing as aetiology, and also further identifying the specific roles played by food handlers in foodborne disease outbreaks throughout Kenya. Strengthening of the food safety regulatory system is also recommended. To prevent further norovirus infections, food handlers found to be norovirus-infected during outbreak investigations should avoid food handling and preparation until such a time they are non-infectious, i.e. at least 72 h’s post-symptom resolution [22]. Excluding food workers while they are symptomatic and until at least 48 h after symptom resolution can prevent further disease transmission. Food safety training should be considered for the study area that includes awareness about norovirus, modes of food borne diseases transmission, and prevention strategies, with an emphasis on hand washing practices. Outbreaks of gastroenteritis should be investigated in the area, and norovirus should be included as a possible cause, among other etiologies.

## Conclusion

The norovirus infection was prevalent amongst food handlers in this informal settlement yet most of them were unaware of the virus. Many food handlers had no medical examination certificates and few were trained on food safety. Infected food handlers had unhygienic practices and some were without knowledge of basic modes of disease transmission. We recommend increased training on hygienic practices to create awareness of norovirus transmission routes.

## Supplementary information

**Additional file 1.** Questionnaire tool.

## Data Availability

The datasets generated during and /or analyzed during the current study are available from the corresponding author on reasonable request. The stool specimens tested were destroyed after one year of preservation as per the Institute of Primate Research (IPR) internal policy.

## Abbreviations

AOR: Adjusted Odd Ratio
CDC: Centers for Disease Control
CHEW: Community Health Extension Workers
CHV: Community Health Volunteers
CI: Confidence interval
CU: Community Unit
DNA: Di-Ribonucleic Acid
IPR: Institute of Primate Research
OR: Odd Ratio
PCR: Polymerase Chain Reaction
RNA: Ribonucleic Acid
