# Supplementary material for: Norovirus infections and knowledge, attitudes and practices in food safety among food handlers in an informal urban settlement, Kenya 2017
Source: BMC Public Health. 2020 Apr 10;20:474. doi: 10.1186/s12889-020-8401-x (PMC7146951; doi:10.1186/s12889-020-8401-x)
Supplement: Supplementary file 1 — Additional file 1. Questionnaire tool. [file 12889_2020_8401_MOESM1_ESM.docx]

**Questionnaire tool**

**PART A: THE FOOD HANDLER CHARACTERISTICS**

**(D1**).What is your age in years? ------------ **(D2).**Gender (1) Male (2) Female___________

**(D3**).What is your highest level of education? (1) None (2) Primary (3) Secondary (4) Tertiary (5) refuse answer

(**D4**).What is your average monthly Income? (1)Income in Kenya shillings.................. (2) Prefer not to answer

**(D5).**What kind of premise is the food handler doing his/her business (1)Permanent building (2) Fixed structures (3)Mobile structure (4) No structure (5) Others (Specify)……………………………….

(**D6**).Have you been sick with diarrhea (Loose stool>3 times per day) in the past one month? (1) No (2) Yes

(**D7**).Have you vomited at any time in the past two weeks? (1) No (2) Yes

**(Q8).**Has anyone in your household had diarrhea (Loose stool>3 times/day) in past one month? (1) No (2) Yes

(**Q9**).Has anyone in your household suffered vomiting illness within the last one month? (1) No (2) Yes

**PART B: KNOWLEDGE, ATTITUDE AND PRACTICE (KNOWLEDGE) (***please read questions as it is written, without further reviewing of each answer with the respondent)*

(**K10)** .When do you think one should wash their hands?

|  |  | (1) No | (2) Yes | (3)Don't know |
| --- | --- | --- | --- | --- |
| i | After visiting toilet |  |  |  |
| ii | After handling money |  |  |  |
| iii | After greeting someone |  |  |  |
| iv | Before food preparation |  |  |  |
| v | Before eating |  |  |  |
| vi | Before cooking food |  |  |  |
| vii | Before serving food |  |  |  |
| viii | Others (Specify).. | | | |

(**K11**). State the advantages of hand washing?

|  |  | (1)No | (2) Yes | (3)Don't know |
| --- | --- | --- | --- | --- |
| i | To prevent self from disease causative organisms |  |  |  |
| ii | To protect others from disease causative organism |  |  |  |
| iii | To remove dirt from hands |  |  |  |
| iv | To attract customers when selling food |  |  |  |
| v | Others (Specify)...... | | | |

(**K12)** .What are some examples of ways that food can be contaminated after cooking?

|  |  | (1) | (2) Yes | (3)Don't know |
| --- | --- | --- | --- | --- |
| i | cockroaches |  |  |  |
| ii | Rats |  |  |  |
| iii | Contaminated water |  |  |  |
| iv | Dirty chopping board |  |  |  |
| v | Aerosols from infected food handler |  |  |  |
| vi | Contaminated environment |  |  |  |
| vii | Flies |  |  |  |
| viii | Others (specify) | | | |

(**K13**). What is the purpose of the medical exam before starting food handling business by food handlers?

|  |  | (1) No | (2) Yes | (3) Don't know |
| --- | --- | --- | --- | --- |
| i | For self-awareness by food handlers |  |  |  |
| ii | As a source of revenue to the government |  |  |  |
| iii | To avoid harassment by public health officers |  |  |  |
| iv | To attract customers |  |  |  |
| v | To obey the law |  |  |  |
| vi | To prevent disease transmission by food handlers |  |  |  |
| Vii | For food handlers to know their disease status |  |  |  |
| Vii | Others (Specify) | | | |

**(K14**) .What are some of the ways that drinking water served to customers can be contaminated?

|  |  | (1) No | | (2) Yes | (3) Don't know |
| --- | --- | --- | --- | --- | --- |
| i | Putting unwashed bare hands into drinking water |  |  | |  |
| ii | After drinking water gets into contact with water used for washing dishes |  |  | |  |
| iii | Sharing of drinking water from the same cup |  |  | |  |
| iv | Spiting on the drinking water by food handlers | (1) No | | (2) Yes | (3) Don't know |
| v | Dust contamination during storage of drinking water |  |  | |  |
| vi | When drinking water gets into contact with sewer water |  |  | |  |
| vii | Others (Specify | | | | |

**(K15).**What diseases are caused by drinking contaminated water (1) typhoid (2) Cholera (3) Worms (4) Amoeba (5) Don't know (6) Others (Specify)_____________________

**(K16)**.Give examples of drinking water treatment methods to make it safe for consumption

|  |  | (1) No | (2) Yes | (3) Don't know |
| --- | --- | --- | --- | --- |
| i | Boiling |  |  |  |
| ii | Adding water guards or aqua guards |  |  |  |
| iii | Filtering |  |  |  |
| iv | Covering the water |  |  |  |
| v | Others (Specify) | | | |

**(K17**).Have you ever heard of Norovirus disease?(0) No (1) Yes.

If Yes how did you know about it?__________________________________________

**(K18)**Why do you think food handlers undergo training on food hygiene safety?

|  |  | (1) No | (2) Yes | (3)Don'tknow |
| --- | --- | --- | --- | --- |
| i | To help improve food safety practices |  |  |  |
| ii | To help acquire catering job |  |  |  |
| iii | To prevent disease outbreak through good practice |  |  |  |
| iv | To increase self esteem |  |  |  |
| v | Others (Specify) | | | |

**ATTITUDE**

**(A19).**Do you think all food handlers should wash their hands?(0) No (1) Yes (2) Don't know

If yes why?_______________________________________________________

If No, why not?____________________________________________________

**(A20**).Are food handlers responsible to protect food sold to customers? (1) Yes (2) No (3) Don't know

If yes why?__________________________________________________

If No, why not?_________________________________________________

**(A21).**Do food handlers need a medical exam before they can sell food? ( (1) Yes (2) No (3) Don't know

If Yes why?______________________________________________________

If No, why not?____________________________________________________

**(A22**).Does the surroundings and environment matter when selling food?(1) No (2) Yes (3) Don't know

If yes why?_____________________________________________________

If No, why not?___________________________________________________

**(A23)**.Should food handlers ensure safe water isserved to customers?(1) No(2) Yes (3) Don't know

If yes why?____________________________________________________

If No, why not?____________________________________________________

**(A24**).Should food handlers worry about diarrhea amongst themselves? (1) No (2) (3)Yes Don't know

If yes why?___________________________________________________

If No, why not?____________________________________________________

(**A25).**Should food handlers be trained on safe food handling practices (1) No(2) Yes (3)Don't know

If yes why?_______________________________________________________

If No, why not?____________________________________________________

**PRACTICE**

(**P26**).What kind of food are you selling now?......(Circle all that apply)

|  | Type of food (YES) | (1) None |
| --- | --- | --- |
| 1 | Animal products only: Sausage, *Mutura, Matumbo,* beef meat, Pork, Fish,  (Others specify...........) |  |
| 2 | Plants products only:Githeri, boiled cereals, *Chapatis, Mandazi*, cabbages  (Others specify...........) |  |
| 3 | Mix of animal and plant products: BEEF STIFF PORRIDGE(Ng'ombeugali), FISH STIFF PORRIDGE(fish ugali), MAIZE AND BEANS (Githeri mix)  (Others specify...........) |  |

(**P27**).Did you prepare the food you are selling now? (1) No (2) Yes

**(P28).** What water source do you use at this point of sale? (1). Rain water (2) River

(3)Water vendors (4) Communal tap water (5) No water used (6) others. Specify________________________________

(**P29)**.How is drinking water dispensed to your customers?(1)cup (2)Jug (3) Tap(4)No water available

(iv). Others. Specify__________________________________

**(P30**).What do you use in washing your hands at the point of sale? (1)Water only (2)Water and soap only (3)Water and sand (4) Sanitizers and water (5) No hand washing done (6) Others (specify)…………………

**(P31**)What do you do to prevent Diarrhea disease when selling food to customers?

|  |  | (1) No | (2) Yes |
| --- | --- | --- | --- |
| i | By not handling food when sick with diarrhea |  |  |
| ii | Avoiding food handling with bare hands |  |  |
| iii | Not allow customers select food with bare hands |  |  |
| iv | Not using same knife to cut raw food and cooked food without washing |  |  |
| v | Washing the cutting board after using between food items |  |  |
| vi | Avoiding blowing into polythene paper to open when serving food in it |  |  |
| vii | Covering food when selling |  |  |
| viii | Taking medication to prevent and treat diarrheal illness |  |  |
| ix | Hand washing regularly |  |  |
| x | Others. Specify | | |

**(P32).**Do you have a valid medical exam certificate? (1) No(2) Yes

If yes can I see it please.(1) Observed (2) Not observed

After how long do you renew you medical certificate?(if observed)______________

**(P33)** Were youTrained as food handler?(1) No (2) Yes

**(P34**).Do you serve drinking water to your food customers?(1) No (2) Yes

If yes can I see it please(1) Observed (2) Not observed

If observed what have you done in the past month for drinking water to make it safe from contamination?(1) Boil water (2) Store in covered jericans (3)Add water guard (4)Filtering (5) Nothing

(6) Others specify………………………………………………………………..

**OBSERVATION**

**(O35).**What type of preparations is the food handler dealing with

|  | Type of preparation | Tick as appropriate |
| --- | --- | --- |
| 1 | Raw |  |
| 2 | Cooked on site |  |
| 3 | Cooked away from selling point |  |

**(O36).** Did the food handler wash his/her hands before or after the interview or observed within 20 minutes?

(2) No(1) Yes

**(O37).**Which observable hand washing services are available where the food handler is working?

|  |  | (2)No | (1) Yes |
| --- | --- | --- | --- |
| i | Water only |  |  |
| ii | Soap and water |  |  |
| iii | Hand drying services |  |  |
| iv | No hand washing services available |  |  |
| v | Others (Specify) | | |

**(O38)**.Did you observe any point where raw food is coming into contact with cooked food? (2) No (1) Yes

If Yes describe _______________________________________________________

**(O39)**.Is the food handler directly touching ready to eat food with bare hands?(2) No (1) Yes

(**O40**).Is the food handler using saliva to ply the polythene paper open?(2)No (1) Yes

**(O41)**. Is the food handler blowing inside polythene papers to open before using them to serve customers? (2) No (1) Yes

**(O42)**.Are/is there dust bins for waste or refuse disposal? (2)No (1)Yes

**(O43)**.Is there drinking water meant for customers? (2) No (1) Yes

If Yes is the water completely covered? (2) No (1) Yes

**(O44)**.Is the ready to eat food covered? (2) No (1) Yes
